# Control of *Francisella* *tularensis* Virulence at Gene Level: Network of Transcription Factors

**DOI:** 10.3390/microorganisms8101622

**Published:** 2020-10-21

**Authors:** Petra Spidlova, Pavla Stojkova, Anders Sjöstedt, Jiri Stulik

**Affiliations:** 1 Department of Molecular Pathology and Biology, Faculty of Military Health Sciences, University of Defence, Trebesska 1575, 50001 Hradec Kralove, Czech Republic; jiri.stulik@unob.cz; 2 Department of Clinical Microbiology, Umeå University, SE-901 85 Umeå, Sweden; pavla.stojkova@gmail.com (P.S.); anders.sjostedt@umu.se (A.S.)

**Keywords:** *Francisella*, virulence, transcription factor, pathogenesis, gene regulation

## Abstract

Regulation of gene transcription is the initial step in the complex process that controls gene expression within bacteria. Transcriptional control involves the joint effort of RNA polymerases and numerous other regulatory factors. Whether global or local, positive or negative, regulators play an essential role in the bacterial cell. For instance, some regulators specifically modify the transcription of virulence genes, thereby being indispensable to pathogenic bacteria. Here, we provide a comprehensive overview of important transcription factors and DNA-binding proteins described for the virulent bacterium *Francisella tularensis*, the causative agent of tularemia. This is an unexplored research area, and the poorly described networks of transcription factors merit additional experimental studies to help elucidate the molecular mechanisms of pathogenesis in this bacterium, and how they contribute to disease.

## 1. Introduction

*Francisella tularensis* belongs to the genus Francisella, which is highly diverse comprising a considerable number of species, and the genus is rapidly expanding. Several species exist globally and many species appear adapted to highly specialized environmental niches and are capable of infecting a broad range of mammals and fish. The species *F. tularensis* encompasses two subspecies pathogenic for humans, *tularensis* and *holarctica*. *F. novicida* is usually described as separate species, although it shares >97% nucleotide identity with *F. tularensis*, the former is a rare human pathogen often derived from environmental sources [1]. However, *F. novicida* is often used as a surrogate for *F. tularensis*, e.g., in genetic studies. *F. tularensis* is a highly infectious human pathogen causing tularemia [2,3]. It is an intracellular pathogen, the virulence of which is based on the ability to escape from phagosomes and replicate in the cytosol of the host cell. Subsequently, after rapid intracellular replication, it subverts the immune system [4,5,6,7,8]. Depending on the route of infection, it may, in severe cases, cause pneumonia [9,10]. Its virulence is controlled at many levels, including molecular signaling, gene transcription, translation, and many posttranslational modifications. Its pathogenicity depends on the production of bacterial virulence factors, the role of which is to attack the host cell and to suppress the immune response.

The most important virulence factors of *F. tularensis* include the capsule [11,12] and surface lipopolysaccharide layer [13,14,15,16]; membrane vesicles [6,17,18]; and secretion systems, especially the Type VI Secretion System (T6SS) [19,20,21,22]. The latter is composed of proteins encoded by genes from the Francisella pathogenicity island (FPI) (*pdpABCDE*, *iglABCDEFGHIJ*, *vgrG*, DotU domain-containing protein (*dotU*)) [20,23,24]. FPI proteins are necessary for Francisella intracellular replication and mutant strains lacking at least one FPI gene are also defective for virulence in vivo [21]. FPI proteins are key components that ensure the ability of *F. tularensis* to modulate phagosome maturation and to escape into the cytosol of the host cell [21], as exemplified by analysis of the best described FPI protein, intracellular growth locus C (IglC) [25]. Interestingly, not all FPI proteins are important for intracellular growth, as reported for IglG [21]; nevertheless, a Δ*iglG* mutant strain exhibited delayed phagosomal escape [26]. FPI gene expression is probably the most important step of Francisella virulence and a detailed understanding of its regulation is essential for the understanding of the *F. tularensis* pathogenesis. 

Last but not least virulence factors are regulatory proteins that control the transcription of virulence genes, mainly the macrophage growth locus, subunit A (MglA)/stringent starvation protein A (SspA)/pathogenicity island gene regulator (PigR) complex [27,28,29,30,31,32], but also numerous others. Many DNA-binding proteins have been identified in Francisella since its complete genome sequencing [24], but only a few of them are well described and reviewed in Reference [33]. This review will be focused on transcription factors that are important for Francisella pathogenesis (Table 1).

## 2. Transcription Factors Involved in Regulation of the FPI 

### 2.1. MglA/SspA Complex Directly Regulates FPI 

One of the best described transcriptional regulators of *F. tularensis* is the MglA protein (macrophage growth locus, subunit A). The protein was identified as necessary for *F. novicida* replication in host cells [34,35] and a mutant strain lacking the *mglA* gene is attenuated in a mouse model of infection. Notably, the mutant does not elicit a protective immune response against infection with the fully virulent parental *F. tularensis* strain [27,28]. MglA is a transcription factor, which regulates up to 100 genes, including the FPI genes [27,28,29]. The most important genes that are associated with *F. tularensis* or *F. novicida* virulence and are known to be positively regulated by MglA are listed in Table 2. 

The MglA protein directly interacts with the SspA protein (Stringent starvation protein A), and this interaction is unique for Francisella genus. The heterodimer MglA/SspA further binds to RNA polymerase (RNAP). In other bacteria, SspA interacts with RNAP as a homodimer [39]. The activity of SspA was especially notable during starvation conditions and its transcription activity is connected with genes that are important and necessary during various stress conditions [28,34]. The regulons of MglA and SspA are similar and both proteins are involved in the regulation of virulence genes in Francisella, including FPI genes. MglA and SspA interact with each other to form a complex with the RNA polymerase, which is essential for activation of the FPI genes [30]. Structural and biochemical analysis showed that MglA is able to form homodimers, but it exhibits a preference for heterodimer formation with SspA [36]. SspA homologs exist in several bacteria and have consistently been associated with virulence [67,68,69,70], whereas MglA is a protein unique for Francisella [36]. In *E. coli*, during the stationary phase, SspA inhibits the level of the global regulator H-NS (histone-like nucleoid structuring), which acts mostly as a repressor, and thus SspA induces stress defense mechanisms, including protection against acid and starvation stress [71]. The importance of MglA for adaptation to oxidative stress was also reported for the *F. tularensis* live vaccine strain (LVS) strain [72].

### 2.2. PigR (FevR) and Guanosine Tsetraphosphate (ppGpp) Interact with the MglA/SspA Complex

Following the discovery of the MglA/SspA regulatory complex, the detailed molecular mechanisms on how this complex interacts with DNA were elucidated. It was shown that FevR (Francisella effector of virulence regulation) is required for expression of FPI genes from the MglA/SspA regulon, and that the expression of *fevR* is upregulated by MglA and FevR/MglA together upregulated virulence gene expression. Subsequently, the importance of FevR for intracellular replication of *F. novicida* and its virulence in vivo was described [31]. A later study confirmed the involvement of FevR in regulating FPI gene expression also in *F. tularensis*, where the ortholog was denoted as PigR. It was confirmed that there was direct interaction between PigR and the MglA/SspA complex and that this interaction was promoted by guanosine tetraphosphate (ppGpp) [32] in the presence of Mg^2+^ [39]. A later study showed that MglA/SspA must be bound to ppGpp to mediate high-affinity interactions with PigR, and that the MglA/SspA complex interacts with the last 22 residues of the PigR-terminal domain [39]. The importance of the interaction between PigR and the MglA/SspA complex has been demonstrated to be necessary for the coordinate control of virulence genes [31,32,37]. Another signal molecule, inorganic polyphosphate (polyP), was identified to be important for MglA/SspA and interactions between target genes and promoters. It was shown that the absence of polyP led to decreased binding of the MglA/SspA complex to the promoters of the pathogenicity determinant protein D (*pdpD*), intracellular growth locus A (*iglA*), *fevR*, and *ppK* genes [40]. Although PigR is able to associate with many promoters, only promoters that contain a specific sequence motif referred to as the PigR-response element (PRE) are positively regulated by PigR/MglA/SspA [38]. 

In *E. coli*, ppGpp is a small regulating molecule that binds directly to the RNA polymerase (RNAP) and could thus both positively and negatively regulate transcription of genes [73]. The level of ppGpp in the bacterial cell is regulated by the ppGpp synthetase (RelA) and a bifunctional protein that is able to synthesize and degrade ppGpp, named SpoT [74]. *relA* and *spoT* genes were identified in the *F. novicida* [75] and also in the *F. tularensis* Schu S4 [24,76] genome. The level of ppGpp in *F. novicida* was affected by a *relA* gene deletion [76], and the deletion of both the *relA* and *spoT* genes led to the complete disappearance of ppGpp [32]. 

*pigR* expression is positively regulated by MigR (macrophage intracellular growth regulator) [32], and MigR was found to be necessary for the alarmone ppGpp accumulation [41]. Moreover, MigR is necessary for effective replication of the LVS strain in macrophages [41]. MigR also regulates the *iglABCD* expression [77], but it lacks a DNA-binding domain, suggesting it does not play a role as a transcription factor and, therefore, its effect on *pigR* and *iglABCD* is indirect. MigR could play a role in the regulation of RelA and SpoT and thereby indirectly influence the ppGpp synthesis [32]. Among others, MigR is involved in the ability of *F. tularensis* to block NADPH oxidase activity in neutrophils [77].

## 3. Regulators Participating in the Oxidative and Nitrosative Stress Responses

### 3.1. OxyR Regulates Genes Involved in the Oxidative Stress Response

The OxyR (hydrogen peroxide-inducible genes activator) transcription factor is a member of the LysR transcriptional regulator family. Similar to other regulatory DNA-binding proteins, members of this family possess the helix-turn-helix (HTH) domain. OxyR is a well-known regulatory protein in *E. coli*, where its activity counteracts the actions of reactive oxygen species and regulates the expression of many genes in response to a variety of stress stimuli [78]. OxyR is important for *F. tularensis* subsp. *holarctica* LVS strain during the oxidative stress response. It was found that the loss of OxyR leads to a lower ability to grow inside macrophages and attenuated virulence in mice [49]. OxyR regulates the expression of the *ahpC*, *katG*, and superoxide dismutase *(sodB)* genes, which act as antioxidant enzymes in Francisella. It is able to bind directly to the promoter of *ahpC* and *katG* [49,50]. Mainly, KatG and OxyR work together and constitute an important system for defense against oxidative stress [79].

### 3.2. Francisella Gene Regulation in the Nitrosative Stress Response

Gene regulation in response to nitrosative stress is poorly studied in *Francisella* species. As mentioned above, AhpC, which is directly regulated by OxyR [49,50], is involved in resistance to reactive nitrogen species (RNS) as reported for the Schu S4 and LVS strains [80]. 

It was also shown that *F. tularensis* is able to change gene expression in response to spermine, a donor of NO molecule (nitrogen monoxide) [81]. Spermine belongs to polyamines that are associated with DNA synthesis, and spermine is produced only by eukaryotic cells [82]. Spermine induces gene expression of Francisella insertion sequences (IS), ISFtu1 and ISFtu2 [81]. IS are small genetic elements that are able to join to multiple DNA sites and thus alter gene expression [83]. Not only that spermine is able to induce the expression of ISFtu1 and ISFtu2 but also the expression of genes downstream to IS element. On the other hand, expression of some Francisella genes can be decreased in response to spermine (*iglA, iglC,* and *iglD*) [81]. 

### 3.3. The RNA-Binding Protein Hfq

The Hfq protein was found to be a necessary host factor required for the synthesis of bacteriophage Qβ RNA in *E. coli* [84]. Hfq is not an ordinary transcription factor due to its preferences to bind RNA rather than DNA. Nevertheless, Hfq was identified as a bacterial nucleoid-associated protein, suggesting it has a role as a transcription factor [85]. An ability to directly bind DNA by its *C*-terminal part was demonstrated in *E. coli* [86]. Hfq is present in the nuclear, cytoplasmic, and membrane fractions in *E. coli* [87], however, its amount varies among the fractions. The largest amounts of Hfq are found in the cytoplasmic and membrane fractions with 30% and 50%, respectively, whereas only 10–20% is present in the nuclear fraction [87]. Its presence close to the DNA chain was also confirmed by electron microscopy [87]. It was suggested that Hfq is a sequence non-specific DNA-binding protein and that the number of binding sites increases with higher concentrations of Hfq [85,86], although a putative Hfq-binding motif has been proposed [86]. Nevertheless, Hfq is predominantly referred to as an RNA-binding protein and plays a key role in the pathogenesis of virulent bacteria such as *Yersinia pseudotuberculosis* [88], *Neisseria meningitides* [89], and *Salmonella enterica* [90]. Hfq is also known for its role in promoting sRNA–mRNA interactions that could be important for the bacterial response to various environment conditions (iron limitation, oxidative stress, anaerobic conditions, glucose starvation) [91]. In support of this hypothesis, Hfq interacts with the sRNA of DsrA (dissimilatory sulfite reductase subunit A) and mRNA of RpoS (RNA polymerase sigma factor), thereby participating in the regulation of the stress response factor RpoS in *E. coli* [92]. 

The Hfq protein is also indispensable in Francisella. Hfq is important for stress tolerance and full virulence in *F. tularensis* subsp. *holarctica*, where it plays a role as a pleiotropic regulator [43] under control of sigma factor 70. It regulates the expression of a multitude of genes, including the FPI genes. Interestingly, Hfq regulates only one (*pdp*) of the two FPI operons (*pdp* and *igl*). Hfq acts, in most cases, as a negative regulator, as suggested by a study at the transcriptomic level in *F. tularensis.* The expression of genes from the *pdp* operon was upregulated at the transcriptomic level in a Δ*hfq* mutant strain, suggesting Hfq is their negative regulator in the wild-type context [43]. A proteomic study revealed decreased levels of the FPI proteins due to an *hfq* gene deletion, which explains why an *hfq* mutant is attenuated [44]. The importance of Hfq in stress tolerance was observed also in *F. novicida* [45].

## 4. Global Regulatory Proteins Important for *F. tularensis* Virulence 

### 4.1. The HU Protein: A Small Unit Necessary for F. tularensis Virulence

Similar to eukaryotic histones, which are responsible for the alteration of the DNA structure and gene regulation, bacteria contain small basic regulatory proteins called histone-like proteins, more precisely nucleoid-associated proteins due to their localization in bacterial cells. It is presumed that they play the same role in the cell as their eukaryotic counterparts [93]. They bind DNA and by bending DNA chains they are able to alter gene expression. Among this group of DNA-binding proteins belongs HU (histone-like factor U), FIS (factor for inversion stimulation), H-NS (histone-like nucleoid structuring), and IHF (integration host factor) proteins [94]. HU and IHF proteins are homologous and are strongly conserved among bacterial species. Additionally, the HU protein enhances the DNA-binding capacity of IHF [95]. Their roles in the cell are indispensable and disruption or malfunction of one of them could lead to the alteration of basic metabolic pathways or even attenuation of virulence of pathogenic bacteria.

Although the HU protein has been well studied in Gram-negative bacteria other than *F. tularensis* [reviewed in Reference 47], its significance for *F. tularensis* subsp. *holarctica* intracellular growth and virulence was elucidated only recently [42]. HU forms heterodimers in most bacteria studied (e.g., Enterobacteriaceae) and is encoded by two genes *hupA* and *hupB* [93], but in some bacteria (e.g., *F. tularensis, M. tuberculosis*) [42,96,97], HU is encoded by a single *hupB* gene and forms homodimers. The HU protein is known to be abundant in the bacterial cell [85], where it is able to influence recombination, replication, transcription, and DNA shape [98,99]. Moreover, the HU protein is capable to modulate the interaction between the IHF nucleoid-associated protein and its target DNA sequence in the *oriC* gene; thereby, HU contributes to DNA replication in *E. coli* [100]. The HU protein binds all nucleic acids in a non-specific manner [101], but it shows higher preferences for abnormal structures, such as four-way junctions or replication forks [102]. By binding to DNA, HU ensures its negative supercoiling, whereby it contributes to the regulation of gene expression [103]. The HU–DNA interaction also provides protection of DNA against free hydroxyl radicals that could irreversibly damage DNA [42,104]. DNA-binding sites of HU are of different lengths in various bacteria [95,99,105,106,107], but preferences for A/T rich sites have been identified [108]. It has been suggested that HU is not a specific transcription regulator, but rather that it acts as a separator of transcription units by its ability of DNA supercoiling, and thus it can influence gene transcription, either as a positive or negative regulator [109].

HU functions as a transcription factor inside the bacterial cell, but it is also secreted extracellularly. In Pseudomonas, HU was found as a component of biofilm formed by the bacterium [110]. It is also secreted by Wolbachia directly into the host cell nucleus, where it binds host DNA [111]. In *F. tularensis* subsp. *holarctica* LVS, HU was found to be secreted since it was identified in the culture filtrate [112] and constituted a minor component of secreted outer membrane vesicles (OMV) [18]. HU is necessary for the *F. tularensis* subsp. *holarctica* FSC200 strain intracellular replication and virulence in mice [42]. It was also proven that it is able to bind dsDNA and, thereby, protect against hydroxyl radicals. Deletion of the *hupB* gene (encoding HU protein) led to the downregulation of PigR and several FPI proteins as well as to the downregulation of *pigR* and several FPI genes. An *F. tularensis* mutant strain lacking *hupB* gene is attenuated in vitro and in vivo as well, demonstrating that HU is important for Francisella virulence [42]. 

Recently, Milanez et al. [113] observed that a *ΔhupAB* mutant of *Salmonella enterica* Enteritidis is highly attenuated and can induce protection against wild-type challenge and thus could be explored as a potential live vaccine. HU proteins are highly conserved among bacterial species [85,96] and it is, therefore, likely that they, as for Salmonella, contribute to virulence in many bacterial species and an *F. tularensis* HU mutant may be a potential live vaccine strain candidate.

### 4.2. AraC, a Novel Global Regulator

The AraC protein (arabinose operon regulatory protein) belongs to the AraC/XylS family that is characterized by the presence of the HTH DNA-binding domain. This domain is conserved in all members of this family and is necessary for the protein’s function as a transcription factor. The evidence of the HTH motif in *F. tularensis* subsp. *holarctica* LVS AraC was confirmed by sequence alignment. Subsequently, a mutant strain lacking the gene encoding AraC was characterized [51]. It was revealed that AraC is necessary for regulation of the oxidative stress response and that AraC is activated only during the oxidative stress conditions. It was also shown that AraC downregulates five FPI genes (pathogenicity determinant protein E (*pdpE*), *pdpC*, *iglJ*, *iglI*, and *dotU*) during oxidative stress, but not during normal physiological conditions. Transcriptome analysis of the mutant strain also revealed the involvement of AraC in the regulation of *aroA* and *rnhA* (heat stress response and homeostasis) during oxidative stress conditions. AraC also plays a crucial role in the regulation of some key components of the TCA cycle during oxidative stress conditions [51]. The connection of the TCA cycle with *F. tularensis* subsp. *holarctica* LVS virulence has been described [114]. Pyruvate dehydrogenase and oxoglutarate dehydrogenase were downregulated in an *araC* deletion mutant strain during oxidative stress conditions [51]. Moreover, AraC regulates the pantothenate synthesis that is a central component of coenzyme A, a molecule necessary for the TCA cycle. It was also observed to be involved in glucose metabolism [51]. Thus, AraC seems to be a global regulator with many functions. AraC could affect RNA polymerase subunits expression, because RpoB and RpoC (RNA polymerase subunit β and β’) were downregulated in the *araC* mutant strain after exposure to various stress conditions, indicating that AraC is a positive regulator of these two proteins. A recent study regarding the AraC describes its possible important role in the pathogenesis of *F. tularensis* subsp. *holarctica* LVS strain. However, there is no proof that there is an AraC–DNA interaction in *F. tularensis* and this needs to be further explored [51]. 

## 5. Regulators Necessary for Regulation of Iron and Metabolism

### 5.1. Ferric Uptake Regulator, Fur

The ferric uptake regulator (Fur) crystal structure was described, and the ability of Fur to bind to promoter sequences containing a Fur box was demonstrated [52]. Fur is a tetrameric protein that is able to bind specific DNA sequences by its splitting into two dimers [52].

Fur is an important component involved in the regulation of iron concentration and contributes to metal homeostasis in the bacterial cell. The concentration of iron is limiting for optimal growth and physiological balance of bacteria, but higher concentrations could be fatal, therefore, it is highly regulated. The activity of Fur and the intracellular concentration of iron affect many key components critical for the virulence of pathogenic bacteria [115,116]. Fur works mainly as a Fe^2+^-dependent transcriptional repressor, but some genes require Fur for their expression [117]. It is necessary for the regulation of several genes involved in the tricarboxylic acid cycle (*frdABD*, *aspA*, *sucCD*, *fumAB*) [118] and superoxide dismutase (SodB) as well [119]. Regulation of iron homeostasis and the functioning of metallo-regulating proteins are crucial for the Francisella physiology [54,55]. The level of host cell iron influences *F. tularensis* subsp. *holarctica* LVS intracellular replication and virulence [120]. On the other hand, if grown under iron-restricted conditions, *F. tularensis* subsp. *holarctica* LVS and *F. tularensis* subsp. *tularensis* Schu S4 were found to produce their own siderophores [53], FslA, which is similar to the polycarboxylate siderophore rhizoferrin [53]. Later, it was found that FslE is also required during iron starvation and could act as a membrane receptor for FslA [121]. The encoding genes belong to *fslABCDEF* operon (also called *fig* operon), which is under Fur control [52] and upregulated during iron starvation [52,53]. Fur is required for full virulence of *F. tularensis* subsp. *holarctica* in both macrophage-like cells and mice [52].

### 5.2. IclR Does Not Contribute Significantly to the F. tularensis Virulence

The IclR family of transcription factors is widely distributed in bacteria and their characteristic feature is the presence of a helix-turn-helix (HTH) domain [122], which allows the protein to bind to the major groove of the DNA chain [123]. The role of the IclR protein for Francisella virulence and pathogenesis has been studied and while IclR is essential for the virulence of *F. novicida* in a mouse model of infection [47], it is dispensable for full virulence of *F. tularensis* subsp. *holarctica* or *F. tularensis* subsp. *tularensis,* despite that the IclR proteins are highly conserved among *Francisella* species [48]. A possible interaction of IclR with RipA at the protein level was identified in *F. tularensis*, suggesting that the RipA membrane protein could affect the IclR transcriptional activity [46].

## 6. Proteins Essential for Gene Transcription

### RNA Polymerase Subunits and Sigma Factors

Critical and absolutely necessary for gene transcription is the RNA polymerase and sigma factors that ensure specific promoter recognizing by the RNA polymerase. The Francisella RNA polymerase is composed of two α subunits, the β, β′ and ω subunits (*rpoA1*, *rpoA2*, *rpoB*, *rpoC*, *rpoZ*, respectively) [30]. Francisella contains two *rpoA* genes that encode two non-identical α subunits, which are able to form both a heterodimer and a homodimer [30,56]. In addition, Francisella contains at least two sigma factors. The major sigma factor σ^70^ is encoded by the *rpoD* gene and an alternative sigma factor, which is a homolog of the σ^32^ heat-shock family proteins, is encoded by the *rpoH* gene [30,57]. Transcripts of several genes were observed to have increased level of expression during heat shock (Hsp40, GroEL, GroES, DnaK, DnaJ, GrpE, ClpB, ClpX, ClpP, and HtpG), and six of them were found to be more abundant during overproduction of RpoH in the live vaccine strain (LVS) (Hsp40, HptG, DnaK, DnaJ, GroES, and GroEL) [57], which are proteins included in the heat stress response and are designated as heat-shock proteins (HSP) [57]. The HSP proteins are under direct regulation of RpoH in *E. coli* [58], suggesting the same model of gene regulation during heat shock conditions in *F. tularensis* subsp. *holarctica* LVS [57]. The response to heat shock is crucial for intracellular bacteria such as Francisella. Most of the RNA-polymerase-regulated genes are required for intracellular growth and full virulence of *F. novicida* [124]. Probably the most important of them is ClpB [125] that directly interacts with DnaK, which is under RpoH control, [59] and a strain defective for the *clpB* gene is an auspicious candidate as a live vaccine strain [126,127]. 

## 7. Response Regulators Involved in Two-Component Systems Work as Transcription Factors

Several important DNA-binding proteins participating in Francisella pathogenicity are also response regulators and parts of two-component systems (TCSs). TCS is usually composed of a membrane-bound sensor histidine kinase and a DNA-binding response regulator. TCS is an important part of the transmission of an external signal to a bacterial cell and the subsequent appropriate response to changing environmental conditions through altering the level of gene expression [128,129]. If the membrane sensor kinase detects an external signal, its phosphorylation occurs. After the transfer of the phosphate group to the response regulator, it is activated and acts as a transcription factor. The response regulator is composed of two domains, a receiver, which is responsible for the phosphate group acceptation, and a DNA-binding domain [130]. So far, only three response regulators have been identified in Francisella, the response regulator PmrA (or its homolog QseB), the response regulator KdpE, and biofilm-regulating Francisella protein regulator BfpR (or homolog in *F. tularensis* FTT_1543) [24]. The occurrence of TCS is different in the *F. tularensis* species and subspecies. Two complete (FTN_1452/FTN_1453 and KdpD/E) and one orphan TCSs (PmrA/QseC) are present in *F. novicida*, whereas no complete TCS was found in *F. tularensis* Schu S4. The sensor kinase FTT_1544 and response regulator KdpE are pseudogenes and FTT_1543, the sensor histidine kinase KdpD, PmrA, and the sensor histidine kinase QseC are orphan TCS components in the Schu S4 strain. *F. tularensis* subsp. *holarctica* FSC200 and LVS have only one response regulator PmrA and one orphan sensor kinase QseC [129,131]. 

### 7.1. QseB/PmrA Response Regulator

QseB, or its homolog PmrA, can be found in all *Francisella* species. Inactivation of *pmrA* gene in *F. novicida* led to reduced survival and growth within human and murine macrophages, and the mutant strain was able to elicit a protective immune response against *F. novicida* wild-type challenge. The first analyses showed that PmrA regulates up to 148 genes, including FPI genes [132,133]. The importance of PmrA in biofilm formation was also described [60]. PmrA is a DNA-binding protein and following phosphorylation, it recognizes its own promoter and also the promoter of the FPI protein PdpD. The histidine kinase that is primarily responsible for phosphorylation of PmrA in *F. novicida* is KdpD [61]. The orphan sensor kinase QseC was also demonstrated to be important for PmrA phosphorylation and Francisella virulence [61]. 

The response regulator PmrA was observed to be important for survival and replication within the macrophages for both *F. tularensis* and *F. novicida*. PmrA works as both a positive and negative regulator. As already mentioned, PmrA participates in *F. novicida* biofilm formation due to the induction of diguanylate cyclase and phosphodiesterase expression. These enzymes are key regulatory components of biofilm formation [60,62]. PmrA is able to bind to the *pdpD* promoter in *F. novicida,* and interaction between PmrA and the MglA/SspA complex was described, suggesting the involvement of PmrA in FPI gene regulation [61]. According to a recent study, PmrA could be a nucleoid-associated protein. It was found that PmrA is able to bind to 252 regions on chromosomal DNA in the *F. tularensis* LVS strain but directly influences only a few of them, mainly as a repressor. PmrA is able to repress the expression of the *priM* gene by direct binding to its promoter, which leads to the ability of *F. tularensis* to grow intracellularly. PriM is referred to as PmrA-repressed inhibitor of intramacrophage growth and it was described as an anti-virulence factor, the expression of which leads to downregulation of intracellular growth [63]. However, the latter was very recently revised [64]. Although, in the first study, PriM was detected as an anti-virulence determinant, because the deletion of the *priM* gene restored an *F. tularensis* subsp. *holarctica* LVS ability to survive within macrophages [63], the second study reported that double deletion mutant strain of *pmrA* (repressor of PriM) and *priM* did not restore the virulence phenotype, suggesting that loss of anti-virulence effect of PriM is caused by an unknown mechanism [64]. 

### 7.2. KdpE 

KdpE, a response regulator transcription factor, interacts with the sensor kinase KdpD in many bacteria, and the KdpE/KdpD system is associated with the cellular regulation of potassium [65]. The presence of KdpE/KdpD was confirmed also in the *F. novicida* genome, but detailed studies are missing. Mutant strains defective in the *kdpE* or *kdpD* genes are attenuated in animal models [47,65]. In other bacteria, the KdpE/KdpD system is responsible for regulating the bacterial response to a variety of stress stimuli, and the DNA-binding activity of KdpE could play a key role in virulence and pathogenesis [65]. 

### 7.3. Biofilm-Regulating Francisella Protein Response Regulator—BfpR

Recently, a response regulator that is involved in biofilm formation in *F. novicida* was characterized and was denoted BfpR (Biofilm-regulating Francisella protein regulator). The presence of two domains, a REC- and a DNA-binding, was confirmed, suggesting it is a typical response regulator with transcription factor activity that is part of a TCS in Francisella. BfpR is encoded by the FTN_1452 gene and it is encoded in an operon with the FTN_1453 sensor kinase, thus, constituting another TCS in Francisella. BfpR has a negative effect on biofilm production, but it is a positive regulator of antimicrobial peptide resistance. The study also suggested that BfpR could be a negative regulator of *iglC* expression [66]. 

## 8. Conclusions

It is clear that Francisella, like other bacteria, possesses a large number of regulatory proteins, the action of which can lead to fine-tuned adaptation to changing environmental conditions. As an important human pathogen, it must be prepared to overcome all of the pitfalls generated by the immune system. Thus, these regulatory proteins might be key components for sustaining Francisella virulence. However, our knowledge is still incomplete and further in-depth studies of these proteins are needed to fully understand the pathogenesis of Francisella and to devise successful strategies to counteract infection with *F. tularensis*.

## Figures and Tables

**Table 1:** Transcription regulators described in this review.

Locus Tag			Gene	Main Function	Reference
Schu S4	LVS	U112			
FTT_1275	FTL_1185	FTN_1290	*mglA*	interacting with SspA, regulation of FPI genes, necessary for intracellular replication	[27,28,29,30,34,35]
FTT_0458	FTL_1606	FTN_0549	*sspA*	interacting with MglA, regulation of FPI genes, necessary for stress response	[28,30,34,36]
FTT_0383	FTL_0449	FTN_0480	*pigR*	interacting with MglA/SspA, regulation of FPI genes	[31,32,37,38,39,40,41]
FTT_0627	FTL_0895	FTN_1054	*hupB*	global regulator, DNA non-specific binding protein	[42]
FTT_0630	FTL_0898	FTN_1051	*hfq*	RNA-binding protein, necessary for stress response, negative regulator of *pdp* operon	[43,44,45]
FTT_0748	FTL_1364	FTN_0720	*iclR*	important only for *F. novicida* virulence in vivo	[46,47,48]
FTT_0556c	FTL_1014	FTN_0959	*oxyR*	regulation of genes involved in oxidative stress response	[49,50]
FTT_1255c	FTL_0689	FTN_1274	*araC*	global regulator, active during oxidative stress conditions, regulation of some FPI genes and tricarboxylic acid (TCA) cycle during oxidative stress	[51]
FTT_0030c	FTL_1831	FTN_1681	*fur*	regulation of iron concentration	[52,53,54,55]
FTT_0350	FTL_0261	FTN_0264	*rpoA1*	RNAP subunit A1, gene transcription	[30,56]
FTT_1442c	FTL_0616	FTN_1412	*rpoA2*	RNAP subunit A2, gene transcription	[30,56]
FTT_0144	FTL_1744	FTN_1568	*rpoB*	RNAP subunit β, gene transcription	[30]
FTT_0145	FTL_1743	FTN_1567	*rpoC*	RNAP subunit β’, gene transcription	[30]
FTT_0703	FTL_1533	FTN_0613	*rpoZ*	RNAP subunit ω, gene transcription	[30]
FTT_1035c	FTL_1050	FTN_0913	*rpoD*	sigma factor 70, major sigma factor	[30,57]
FTT_1112c	FTL_0851	FTN_1092	*rpoH*	sigma factor 32, regulation of heat shock proteins	[30,57,58,59]
FTT_1557c	FTL_0552	FTN_1465	*pmrA/qseB*	response regulator, important for biofilm formation in *F. novicida*, intracellular replication, interacting with MglA/SspA	[60,61,62,63,64]
FTT_1735c (pseudo)	-	FTN_1714	*kdpE*	response regulator, regulation of potassium	[47,65]
FTT_1543	-	FTN_1452	*bfpR*	positive regulator of antimicrobial peptide resistance, regulation of biofilm in *F. novicida*	[66]

**Table 2:** Genes that are associated with *Francisella tularensis* or *Francisella novicida* virulence and are positively regulated by macrophage growth locus, subunit A (MglA).

Locus Tag	Gene	Description	Reference
FTT_1344	*pdpA*	FPI, Pathogenicity determinant protein A	[28]
FTT_1345 (FTN_1310)	*pdpB*	FPI, Pathogenicity determinant protein B	[28,29]
FTT_1346	*iglE*	FPI, Intracellular growth locus E	[28]
FTT_1347	*vgrG*	FPI, Valine-glycine repeat protein G	[28]
FTT_1348	*iglF*	FPI, Intracellular growth locus F	[28]
FTT_1349	*iglG*	FPI, Intracellular growth locus G	[28]
FTT_1350	*iglH*	FPI, Intracellular growth locus H	[28]
FTT_1351	*dotU*	FPI, DotU domain-containing protein	[28]
FTT_1352	*iglI*	FPI, Intracellular growth locus I	[28]
FTT_1353	*iglJ*	FPI, Intracellular growth locus J	[28]
FTT_1354	*pdpC*	FPI, Pathogenicity determinant protein C	[28]
FTT_1355	*pdpE*	FPI, Pathogenicity determinant protein E	[28]
FTT_1356c	*iglD*	FPI, Intracellular growth locus D	[28]
FTT_1357c (FTN_1322)	*iglC*	FPI, Intracellular growth locus C	[28,29]
FTT_1358c (FTN_1323)	*iglB*	FPI, Intracellular growth locus B	[28,29]
FTT_1359c (FTN_1324)	*iglA*	FPI, Intracellular growth locus A	[28,29]
FTT_1360c (FTN_1325)	*pdpD*	FPI, Pathogenicity determinant protein D	[28,29]
FTN_1051	*hfq*	Host factor for bacteriophage Qβ RNA replication	[29]
FTN_0549	*sspA*	Stringent starvation protein A	[29]
FTN_1054	*hupB*	DNA-binding protein HU-beta	[29]
